# Short‐Term Outcomes and Sex‐Based Analysis Following Chest Pain Presentations to Emergency Departments in Western Australia—An AUS‐MOCHA Substudy

**DOI:** 10.1111/1742-6723.70235

**Published:** 2026-02-19

**Authors:** Jonathon Stewart, Juan Lu, Adrian Goudie, Frank M. Sanfilippo, Girish Dwivedi

**Affiliations:** ^1^ Medical School, The University of Western Australia Crawley Western Australia Australia; ^2^ Harry Perkins Institute of Medical Research Murdoch Western Australia Australia; ^3^ Community and Virtual Care, East Metropolitan Health Service Perth Western Australia Australia; ^4^ Department of Emergency Medicine Fiona Stanley Hospital Murdoch Western Australia Australia; ^5^ School of Population and Global Health, University of Western Australia Crawley Western Australia Australia; ^6^ Department of Cardiology Fiona Stanley Hospital Murdoch Western Australia Australia; ^7^ Victor Chang Cardiac Research Institute Sydney New South Wales Australia

**Keywords:** acute coronary syndrome, chest pain, NSTEMI, sex, STEMI, triage, troponin, unstable angina

## Abstract

**Objective:**

To describe emergency department (ED) chest pain presentations and outcomes, and sex‐based differences in Western Australia.

**Methods:**

We conducted a retrospective cohort study using linked ED, hospital, morbidity, mortality, and pathology data from the Western Australian Data Linkage System as part of the AUS‐MOCHA project. We included the index presentation for adults presenting with non‐traumatic chest pain between January 2016 and October 2020. Comorbidities were identified via a 10‐year lookback of linked morbidity data. The primary outcome was diagnosis of acute coronary syndrome (ACS) or all‐cause death within 30 days of ED discharge.

**Results:**

The study included 64,404 patients (mean age 54.8 years, 49.2% female). Most presented ‘out‐of‐hours’ (62.2%) and received an Australasian Triage Scale (ATS) Category of 2 (85.2%). Females were less likely to receive an ATS 1 and more likely to receive ATS 3 or 4. This difference persisted after propensity matching. However, males were more comorbid and had a higher incidence of 30‐day outcomes than females. There was no statistically significant difference in ATS categories between males and females when assessing a propensity matched cohort of only those who experienced 30‐day outcome. Among patients discharged home from the ED, 0.4% were diagnosed with ACS and 0.1% died within 30 days.

**Conclusion:**

Patients who were discharged home from EDs had a low risk of subsequent ACS or death within 30 days. Females were triaged less urgently than males, yet had lower 30‐day ACS and death rates. Among patients with 30‐day outcomes, triage scores did not differ by sex.

## 1 Introduction

Chest pain is one of the most common presenting symptoms to Emergency Departments (EDs) [1]. In Australia in 2022–23, there were 427,699 chest pain presentations to EDs, accounting for 4.9% of all ED presentations [1]. Causes of chest pain range from benign to life‐threatening. Acute coronary syndromes (ACS) (consisting of myocardial infarction (MI) and unstable angina (UA)) are potentially life‐threatening causes of chest pain [2]. Therefore, rapid risk stratification is important [2]. To help achieve this, there are well established guidelines and clinical care standards [2, 3].

The Australasian College for Emergency Medicine (ACEM) recommends that chest pain of likely cardiac nature is triaged as ‘Category 2’ on the Australasian Triage Scale (ATS), with assessment and treatment commenced within 10 min of presentation [4]. Internationally, there have been reported sex‐based differences in the triage of chest pain, with women being less likely to be triaged as emergent, experience longer wait times, and be less likely to be admitted [5]. Chest pain protocols for women have been developed in attempts to reduce these inequalities; however, they are not in widespread clinical use [6]. Presentation time may influence outcomes, with out‐of‐hours presentations being associated with higher seven‐day mortality [7].

Serial sampling of cardiac specific troponin at clearly defined periods after presentation is an important component of ACS risk stratification and diagnosis [2]. High sensitivity troponin assays (hs‐cTn) have allowed elevated or rising troponin levels to be detected earlier and their use has become increasingly widespread [2, 8]. Rapid assessment protocols using troponin at presentation and at 1 or 2 h have been validated, however the optimal risk stratification pathway is an area of ongoing research [2, 9, 10].

We conducted a population‐based cohort study using linked administrative health data from the AUS‐MOCHA research project to provide a descriptive analysis of chest pain presentations and outcomes in Western Australia (WA) [11]. We also assessed differences in outcomes by sex and ‘out‐of‐hours’ presentation to EDs.

## 2 Methods

WA has a population of almost three million as of June 2024 (10.9% of the Australian population), with most residing in the Perth metropolitan area [12]. The WA population is representative of the Australian population in terms of key demographics, social and health economic indicators [13]. WA has three adult tertiary hospitals and multiple regional centres. WA also has a longstanding and comprehensive data linkage service, the Western Australian Data Linkage System (WADLS) [14]. This system is able to accurately link state‐wide emergency department data with laboratory, morbidity (hospital admissions) and mortality data [14].

### 2.1 Data Source and Study Cohort

Methods used to create the study dataset have been previously described in detail [14]. Data from the WADLS were obtained for the period 1 January 2016 to 24 October 2020. Core statewide datasets of the WADLS held and dynamically linked by WA Health include ED presentations (including ATS category), hospital admissions (public and private) and deaths. Linkage is based on probabilistic matching of demographic data (such as unit medical record number, first and last name, address, date of birth, and sex), with uncertain links being checked manually. The data were additionally linked with pathology test results from PathWest Laboratory Medicine, which provides pathology services to Western Australian public hospitals. Invalid troponin results within the pathology dataset (reported as lipaemic, insufficient sample, haemolysed or cancelled) were discarded. We included all valid troponin test results between ED presentation time and ED discharge time. All sites used the same high sensitivity troponin test (hsTnI) throughout the study period. Troponin was positive if the result was ≥ 16 ng/L for females and ≥ 26 ng/L for males. Past medical history of cardiovascular risk factors was identified through a 10‐year lookback (from date of index presentation) using data from linked hospital admissions (Appendix S1).

The initial AUS‐MOCHA dataset contained 374,766 records (ED presentations) for 65,953 unique patients. This was filtered to include only patients presenting to the ED with non‐traumatic chest pain (Appendix S2), resulting in 130,320 records for 64,432 patients. Patients under 18 were excluded and only the index (first‐in‐period) presentation was considered. The final dataset contained 64,404 records for 64,404 patients. Presentation date/time, investigation date/time, and discharge date/time variables were used to calculate time from presentation to first troponin test, time between first and second troponin test, and ED length of stay.

Within the ED data, an ED admission ‘in‐hours’ was defined as an ED presentation time between 08:00 and 17:00, Monday to Friday. All other presentation times were considered ‘out‐of‐hours’. ED discharge destination included values of: (i) Home (which included patients discharged back to a nursing home); (ii) Admitted (patients admitted to a hospital ward, mental health ward, ED Observation Ward or transferred to another hospital); (iii) Did not wait/Left at own risk; (iv) Died; (v) Unknown/Other.

### 2.2 Outcomes

The primary outcome was diagnosis of ACS or all‐cause death within 30 days of ED discharge, with ACS being defined as the composite of ST‐segment elevation myocardial infarction (STEMI) or non‐ST‐segment elevation myocardial infarction (NSTEMI), and unstable angina, identified through linked morbidity data using ICD‐10‐AM codes (Appendix S3) [15].

### 2.3 Statistical Analysis

All analyses were completed in Python (version 3.10.12). Comparisons were made between those who had an outcome within 7‐ and 30 days of discharge, and those who did not. Categorical variables are reported as counts and percentages, with comparisons using the Pearson chi‐squared test. Continuous variables are reported as mean and standard deviation with comparisons using the Mann–Whitney *U* test. A *p*‐value of less than 0.05 was considered statistically significant. Male and female cohorts were propensity matched based on their age and comorbidities to assess the impact of sex on triage score and outcome. Details of propensity matching are provided in Appendix S4.

### 2.4 Ethics Approval

This project received ethics and governance approval from the WA Department of Health Human Research Ethics Committee and Research Governance Office (RGS0000002876) and the Human Research Ethics Committee of The University of Western Australia (2021/ET000538).

## 3 Results

### 3.1 Demographics

Demographics are described in Table 1. The mean age of the cohort was 54.8 years (SD 18.9, range 18–106). Females accounted for 49.2%, and Indigenous people accounted for 3.9% of the cohort. The majority of patients (62.2%) presented ‘out‐of‐hours’.

**TABLE 1:** Study cohort characteristics.

Characteristic	Count (%)						
	Total cohort	Within 7 days of index visit discharge			Within 30 days of index visit discharge		
		Diagnosis of ACS or death	No diagnosis of ACS or death	*p*	Diagnosis of ACS or death	No diagnosis of ACS or death	*p*
Number of patients	64,404	4046 (6.3)	60,358 (93.7)		4429 (6.9)	59,975 (93.1)	
Demographics							
Sex							
Male	32,659 (50.7)	2796 (69.1)	29,863 (49.5)	< 0.001	3017 (68.1)	29,642 (49.4)	< 0.001
Female	31,713 (49.2)	1250 (30.9)	30,463 (50.5)		1411 (31.9)	30,302 (50.5)	
Other/Not stated	32 (0.05)	0 (0)	32 (0.05)		1 (0.02)	31 (0.05)	
Age (years), mean (Standard deviation)	54.8 (18.9)	67.5 (13.8)	53.9 (18.9)	< 0.001	67.8 (13.9)	53.8 (18.9)	< 0.001
Age range (years)	18–106	20–102	18–106		20–102	18–106	
Indigenous	2547 (3.9)	145 (3.6)	2402 (4.0)	0.23	171 (3.9)	2376 (4.0)	0.75
Time of day of ED presentation							
‘In‐hours’	24,338 (37.8)	1448 (35.8)	22,890 (37.9)	0.009	1595 (36.0)	22,743 (37.9)	0.015
‘Out‐of‐hours’	40,066 (62.2)	2598 (64.2)	37,468 (62.1)		2834 (64.0)	37,232 (62.1)	
Triage score							
1	949 (1.5)	376 (9.3)	573 (0.9)	< 0.001	398 (9.0)	551 (0.9)	< 0.001
2	54,871 (85.2)	3564 (88.1)	51,307 (85.0)		3888 (87.8)	50,983 (85.0)	
3	6686 (10.4)	98 (2.4)	6588 (10.9)		129 (2.9)	6557 (10.9)	
4	1814 (2.8)	8 (0.2)	1806 (3.0)		14 (0.3)	1800 (3)	
5	84 (0.1)	0 (0)	84 (0.1)		0 (0)	84 (0.1)	
Troponin tests							
Troponin test done							
No	36,052 (56.0)	2362 (58.4)	33,690 (55.8)	0.002	2619 (59.1)	33,433 (55.7)	< 0.001
Yes	28,352 (44.0)	1684 (41.6)	26,668 (44.2)		1810 (40.9)	26,542 (44.3)	
Average time to first troponin test (Minutes, [Standard deviation])	49.7 (67.5)	46.8 (51.3)	49.9 (49.0)	< 0.001	46.9 (50.7)	49.9 (49.0)	< 0.001
First Troponin test result							
Positive	2943 (10.4)	965 (57.3)	1978 (7.4)	< 0.001	1007 (55.6)	1936 (7.3)	< 0.001
Negative	25,409 (89.6)	719 (42.7)	24,690 (92.6)		803 (44.4)	24,606 (92.7)	
Second troponin test							
Not done	22,094 (77.9)	1207 (71.7)	20,887 (78.3)	< 0.001	1303 (72.0)	20,791 (78.3)	< 0.001
Done	6258 (22.1)	477 (28.3)	5781 (21.7)		507 (28.0)	5751 (21.7)	
Average time from first to second troponin test (Minutes, [Standard deviation])	152.1 (67.5)	151.0 (66.9)	152.2 (67.5)	0.73	151.2 (66.5)	152.2 (67.6)	0.64
Second troponin test result							
Positive	1173 (18.7)	361 (75.7)	812 (14.1)	< 0.001	376 (74.2)	797 (13.9)	< 0.001
Negative	5085 (81.3)	116 (24.3)	4969 (85.9)		131 (25.8)	4954 (86.1)	
ED Length of stay							
Mean length of stay (hours)	3.7	3.8	3.7	0.0017	3.9	3.7	< 0.001
Length of stay < 4 h	45,481 (70.6)	2595 (64.1)	42,886 (71.1)		2814 (63.5)	42,667 (71.1)	
ED departure destination							
Home	28,087 (43.6)	57 (1.4)	28,030 (46.4)	< 0.001	117 (2.6)	27,970 (46.6)	< 0.001
Admitted	33,572 (52.1)	3690 (91.2)	29,882 (49.5)		3984 (90.0)	29,588 (49.3)	
Did not wait/Left at own risk	345 (0.5)	5 (0.1)	340 (0.6)		6 (0.1)	339 (0.6)	
Died	18 (0.03)	18 (0.4)	0 (0)		18 (0.4)	0 (0)	
Unknown/Other	2382 (3.7)	294 (7.3)	2088 (3.5)		322 (7.3)	2060 (3.4)	
Comorbidities							
ACS	6905 (10.7)	980 (24.2)	5925 (9.8)	< 0.001	1107 (25.0)	5798 (9.7)	< 0.001
Chest pain	11,275 (17.5)	743 (18.4)	10,532 (17.5)	0.15	858 (19.4)	10,417 (17.4)	0.001
Heart failure	3052 (4.7)	282 (7.0)	2770 (4.6)	< 0.001	361 (8.2)	2691 (4.5)	< 0.001
Atrial fibrillation	4668 (7.3)	353 (8.7)	4315 (7.2)	< 0.001	419 (9.5)	4249 (7.1)	< 0.001
Hypertension	10,337 (16.1)	1230 (30.4)	9107 (15.1)	< 0.001	1398 (31.6)	8939 (14.9)	< 0.001
Diabetes	8042 (12.5)	909 (22.5)	7133 (11.8)	< 0.001	1033 (23.3)	7009 (11.7)	< 0.001
Chronic kidney disease	2328 (3.6)	269 (6.7)	2059 (3.4)	< 0.001	326 (7.4)	2002 (3.3)	< 0.001
Cerebrovascular disease	2551 (4.0)	288 (7.1)	2263 (3.8)	< 0.001	326 (7.4)	2225 (3.7)	< 0.001
COPD	3195 (5.0)	217 (5.4)	2978 (4.9)	0.24	280 (6.3)	2915 (4.9)	< 0.001

*Note:* Cohort characteristics stratified by outcome (ACS or death). Within x days of index visit means ≤ x days from index visit discharge time. In‐hours = 08:00–17:00, Monday to Friday. Out‐of‐hours = 00:00–07:59, and 17:01–24:00 Monday to Friday, and any time on Saturday or Sunday. *p*‐value compares Outcome to No outcome group. Departure destination ‘Home’ includes discharge back to nursing home/hostel. Departure destination ‘Admitted’ includes admission to ward, mental health unit, ED observation ward, or transfer to other hospital. Departure destinations for cohort ‘Did not wait’ *n* = 57 and ‘Left at own risk’ *n* = 288. Acute Coronary Syndrome, defined as the composite of ST‐segment elevation myocardial infarction (STEMI) or non‐ST‐segment elevation myocardial infarction (NSTEMI), and unstable angina, identified through linked morbidity data using ICD‐10‐AM codes (Appendix S3).

Abbreviations: ACS, acute coronary syndrome; COPD, chronic obstructive pulmonary disease; ED, emergency department; Index visit, date of discharge from ED index visit; Time to troponin test, time to troponin test being taken; Triage score, Australasian Triage Scale Category [4].

In the total cohort, male and female patients were of similar age (mean age 54.3 vs. 55.3), had a similar proportion of in‐hours presentations (37.2% vs. 38.4%), and identical length of stay (3.7 vs. 3.7 h) (Table 2). There were slightly more troponin tests performed in the male cohort (44.9% vs. 43.1%). The total male cohort had more comorbidities, with a higher prevalence of ACS (13.0% vs. 8.4%), heart failure (5.0% vs. 4.5%), atrial fibrillation (8.0% vs. 6.5%), hypertension (17.1% vs. 15.0%), diabetes (13.5% vs. 11.5%), chronic kidney disease (4.2% vs. 3.0%) and cerebrovascular disease (4.3% vs. 3.7%). The female cohort had a higher prevalence of chest pain (18.9% vs. 16.2%) and chronic obstructive pulmonary disease (5.5% vs. 4.4%).

**TABLE 2:** Study cohort characteristics by sex.

	Cohort	Total			30‐day outcome		
		Male	Female	*p*	Male	Female	*p*
Number of patients	64,404	32,659	31,713		3017	1411	
Demographics							
Age (years), mean (Standard deviation)	54.8 (19.0)	54.3 (18.3)	55.3 (19.5)	< 0.001	65.6 (13.3)	72.4 (14.1)	< 0.001
Age range (years)	18–106	18–102	18–106		25–101	20–102	
Indigenous	2547 (4.0)	1164 (3.6)	1383 (4.4)		98 (3.3)	73 (5.2)	
Time of day of ED presentation							
‘In‐hours’	24,338 (37.8)	12,162 (37.2)	12,163 (38.4)		1079 (35.8)	516 (36.6)	
‘Out‐of‐hours’	40,066 (62.2)	20,497 (62.8)	19,550 (61.7)		1938 (64.2)	895 (63.4)	
Triage score							
1	949 (1.5)	666 (2.0)	282 (0.9)	< 0.001	294 (9.7)	103 (7.3)	< 0.001
2	54,871 (85.2)	27,964 (85.6)	26,879 (84.8)		2639 (87.5)	1249 (88.5)	
3	6686 (10.4)	3106 (9.5)	3577 (11.3)		79 (2.6)	50 (3.5)	
4	1814 (2.8)	876 (2.7)	938 (3.0)		5 (0.2)	9 (0.6)	
5	84 (0.1)	47 (0.1)	37 (0.1)		0 (0)	0 (0)	
Troponin test							
Troponin test done							
No	36,052 (56.0)	17,998 (55.1)	18,041 (56.9)		1756 (58.2)	862 (61.1)	
Yes	28,352 (44.0)	14,661 (44.9)	13,672 (43.1)		1261 (41.8)	549 (38.9)	
Average time to first troponin test (Minutes, [Standard deviation])	49.7 (67.5)	48.0 (48.1)	51.6 (50.2)	< 0.001	45.3 (50.1)	50.7 (51.8)	< 0.001
First Troponin test result							
Positive	2943 (10.4)	1765 (12.0)	1188 (13.8)		675 (53.5)	332 (60.5)	
Negative	25,409 (89.6)	12,896 (88.0)	12,484 (86.2)		586 (46.5)	217 (39.5)	
Second troponin test							
Not done	22,094 (77.9)	11,293 (77.0)	10,782 (78.9)		920 (73.0)	383 (69.8)	
Done	6258 (22.1)	3368 (23.0)	2890 (21.1)		341 (27.0)	166 (30.2)	
Average time from first to second troponin (Minutes, [Standard deviation])	152.1 (67.5)	151.6 (63.9)	152.7 (71.4)	0.81	148.4 (62.1)	156.9 (74.6)	0.15
Second troponin test result							
Positive	1173 (18.7)	651 (2.0)	522 (1.7)		243 (8.1)	133 (9.4)	
Negative	5085 (81.3)	2717 (8.3)	2368 (7.5)		98 (3.2)	33 (2.3)	
ED Length of stay							
Mean length of stay	3.7	3.7	3.7	0.01	3.8	4.2	< 0.001
Length of stay < 4 h	45,481 (70.6)	22,943 (70.3)	22,512 (71.0)		1997 (66.2)	816 (57.8)	
ED departure destination							
Home	28,087 (43.6)	13,501 (41.3)	14,576 (46.0)		76 (2.5)	41 (2.9)	
Admitted	33,572 (52.1)	17,525 (53.7)	16,026 (50.5)		2696 (89.1)	1288 (90.5)	
Did not wait/left at own risk	345 (0.5)	196 (0.6)	149 (0.5)		5 (0.2)	< 5 (< 0.4)	
Died	18 (0.03)	10 (0.03)	8 (0.03)		10 (0.3)	8 (0.6)	
Unknown/other	2382 (3.7)	1427 (4.4)	954 (3.0)		240 (7.9)	81 (5.7)	
PMHx							
ACS	6905 (10.7)	4244 (13.0)	2659 (8.4)	< 0.001	754 (25.0)	353 (25.0)	0.99
Chest pain	11,275 (17.5)	5288 (16.2)	5984 (18.9)	< 0.001	524 (17.4)	334 (23.7)	< 0.001
Heart failure	3052 (4.7)	1621 (5.0)	1431 (4.5)	< 0.01	198 (6.6)	163 (11.5)	< 0.001
Atrial fibrillation	4668 (7.3)	2616 (8.0)	2051 (6.5)	< 0.001	253 (8.4)	166 (11.8)	< 0.001
Hypertension	10,337 (16.1)	5585 (17.1)	4751 (15.0)	< 0.001	866 (28.7)	532 (37.7)	< 0.001
Diabetes	8042 (12.5)	4397 (13.5)	3642 (11.5)	< 0.001	672 (22.3)	361 (25.6)	0.013
Chronic kidney disease	2328 (3.6)	1373 (4.2)	954 (3.0)	< 0.001	205 (6.8)	121 (8.6)	0.035
Cerebrovascular disease	2551 (4.0)	1390 (4.3)	1161 (3.7)	< 0.001	196 (6.5)	130 (9.2)	0.001
COPD	3195 (4.0)	1445 (4.4)	1748 (5.5)	< 0.001	157 (5.2)	123 (8.7)	< 0.001

*Note:* Count (%) by cohort. Cohort characteristics by sex and 30‐day outcome (ACS or death). The total of male and female does not sum to number in the total cohort due to patients of other/not stated sex who are not included in this table. Within x days of index visit means ≤ x days from index visit discharge time. In‐hours = 08:00–17:00, Monday to Friday. Out‐of‐hours = 00:00–07:59, and 17:01–24:00 Monday to Friday, and any time on Saturday or Sunday. *p*‐value compares Outcome to No outcome group. Departure destination ‘Home’ includes discharge back to nursing home/hostel. Departure destination ‘Admitted’ includes admission to ward, mental health unit, ED observation ward, or transfer to other hospital. Departure destinations for cohort ‘Did not wait’ *n* = 57 and ‘Left at own risk’ *n* = 288.

Abbreviations: ACS, acute coronary syndrome; COPD, chronic obstructive pulmonary disease; ED, emergency department; Index visit, date of discharge from ED index visit; Time to troponin test, time to troponin test being taken; Triage score, Australasian Triage Scale Category [4].

### 3.2 Triage

Most patients received a triage score of two (85.2%), with a statistically significant association between sex and triage score (Table 2), with females being less likely than males to receive a triage score of one and more likely to receive a triage score of three or four. This association was unchanged following propensity matching and remained statistically significant (Table 3). A similar proportion of male and female patients received a triage score of two (85.6% and 84.8%, respectively) and five (0.1% and 0.1%). Males with a triage score of one, two, or three were more likely to have an outcome within 30 days than females with the same triage score. There was no statistically significant difference in triage scores between males and females when assessing a propensity matched cohort of only those who experienced 30‐day outcome (Table 3).

**TABLE 3:** Propensity matched triage score by sex.

	Total cohort				30‐day outcome			
	Total	Male	Female	*p*	Total	Male	Female	*p*
Number of patients	59,274	29,637	29,637		2426	1213	1213	
Triage score								
1	867 (1.5)	611 (2.1)	256 (0.9)	**< 0.001**	193 (8.0)	100 (8.2)	93 (7.7)	**0.14**
2	50,416 (85.1)	25,324 (85.5)	25,092 (84.7)		2161 (89.1)	1085 (89.5)	1076 (88.7)	
3	6230 (10.5)	2849 (9.6)	3381 (11.4)		62 (2.6)	26 (2.1)	36 (3.0)	
4	1685 (2.8)	810 (2.7)	875 (3.0)		10 (0.4)	2 (0.2)	8 (0.7)	
5	76 (0.1)	43 (0.2)	33 (0.1)		0 (0)	0 (0)	0 (0)	

*Note:* Propensity matched triage score by sex for total cohort and 30‐day outcome (ACS or death) cohort.

Abbreviation: Triage score, Australasian Triage Scale category [4].

### 3.3 Troponin

Troponin results are described in Tables 1 and 2. One or more troponin tests were performed for 28,352 (44.0%) patients. Of these, the average time from presentation to first troponin test being taken was 49.7 min. First troponin was negative in 25,409 (89.6%) patients and positive in 2943 (10.4%) patients. The average time from first troponin test to second troponin test was 152.1 min. There was a small but statistically significant difference in average time from presentation to first troponin test for those with 30‐day outcome (46.9 vs. 49.9 min). A second troponin test was performed for 6258 patients and was positive in 1173 patients (18.7%). A second troponin test was positive in 217 patients (3.5%) who had a negative first troponin test. There was no significant difference in time from first to second troponin test between outcome groups.

### 3.4 Length of Stay

The mean length of stay in the ED, measured from the time of triage to the time of discharge, was 3.7 h and 70.6% of patients had an ED length of stay less than 4 h (Table 1). Length of stay was less than 4 h for 62.9% of admitted patients and 79.1% of discharged patients. Length of stay was less than 8 h for 93.4% of admitted patients and 98.1% of discharged patients (Table S2).

### 3.5 Departure Destination

Just over half of all patients (52.1%) were admitted, 43.6% were discharged home, and 0.5% either ‘did not wait’ or left at their own risk. There were 18 patients (0.03%) who died in ED. Of those with a 30‐day outcome, 90% were admitted at their index presentation, 2.6% were discharged home, and 7.3% had an unknown or ‘other’ departure destination.

### 3.6 Outcomes

Compared to the total cohort, those with an outcome within 30 days of index visit were older (67.8 vs. 53.8), more likely to present out‐of‐hours (64.0% vs. 62.1%), and receive a higher priority triage score (Table 1). They were also more likely to have troponin tests, and a positive troponin result. They were more likely to be admitted (90% vs. 50.9%) and have a medical history that included any of the assessed comorbidities. There was no statistically significant difference in Indigenous status between whole cohort and 30‐day outcome group. Of those who presented out‐of‐hours, 7.1% experienced 30‐day outcome, compared to 6.6% of those who presented in‐hours. This difference was statistically significant. Males were significantly more likely than females to have a 30‐day outcome (9.2% vs. 4.5%), and the male 30‐day outcome cohort were younger (65.6 vs. 72.4) (Table 2). In the propensity matched cohort, 9.0% of males and 4.4% of females experienced 30‐day outcomes (Table 3).

Outcomes for 7 and 30‐day follow‐up for the total cohort are provided in Table 4 and Table S1. Within 7 days of index presentation, 218 patients (0.3%) had died, 3959 (6.2%) had been diagnosed with ACS, and 4091 (6.4%) had represented to the ED. Of those who represented to ED, 843 (1.3% of initial cohort) had a presenting complaint of chest pain. Within 30 days of presentation to ED, 432 patients (0.7%) had died, 4198 (6.5%) had been diagnosed with ACS, and 9510 (14.8%) had represented to the ED. Of those who represented to ED, 1677 (2.6% of initial cohort) had a presenting complaint of chest pain.

**TABLE 4:** Study cohort outcomes (within 30 days of discharge from index visit).

Outcome	Total cohort (*n* = 64,404)	Admitted (*n* = 33,572)	Discharged home (*n* = 28,087)	Did not wait/left at own risk (*n* = 345)
ACS or death	4429 (6.9)	3984 (11.9)	117 (0.4)	6 (1.7)
Death	432 (0.7)	364 (1.1)	24 (0.1)	0 (0)
ACS	4198 (6.5)	3769 (11.2)	105 (0.4)	6 (1.7)
STEMI	877 (1.4)	698 (2.1)	6 (0.02)	0 (0)
NSTEMI	2352 (3.7)	2200 (6.6)	48 (0.2)	0 (0)
Unstable angina	1021 (1.6)	919 (2.7)	53 (0.2)	6 (1.7)
Represented to ED	9510 (14.8)	5442 (16.2)	3290 (11.7)	79 (23.0)
Represented to ED with chest pain	1677 (2.6)	942 (2.8)	622 (2.2)	9 (2.6)

*Note:* Count (percent of sub cohort). Cohort outcomes within 30 days of discharge from index visit according to discharge location. ACS, diagnosis of STEMI, or NSTEMI, or ACS (see Appendix S3). Transfer to another ED is not counted as representation. Representation with chest pain is based on presenting symptom at subsequent ED presentation. Within x days of index visit means date of outcome ≤ 30 days from time of index visit ED discharge. Counts of admitted, discharged, and left at own risk do not add up to count of total cohort as there are also other discharge destinations (Died = 18, Unknown/other = 2382). Departure destination ‘Home’ includes discharge back to nursing home/hostel. Departure destination ‘Admitted’ includes admission to ward, mental health unit, ED observation ward, or transfer to other hospital. Departure destinations for cohort ‘Did not wait’ *n* = 57 and ‘Left at own risk’ *n* = 288.

Abbreviations: ACS, acute coronary syndrome; ED, emergency department; NSTEMI, non‐ST‐segment elevation myocardial infarction; STEMI, ST‐segment elevation myocardial infarction.

### 3.7 Discharge Home From ED

Details of patients who were discharged home from ED, and of those who left at their own risk are also described in Table 4. Within 7 days of ED discharge 53 patients (0.2%) had been diagnosed with ACS, and nine (0.03%) died. Within 30 days of ED discharge, 105 (0.4%) patients had a diagnosis of ACS and 24 (0.1%) had died. In those patients who left at their own risk, there were no deaths and six (1.7%) diagnoses of ACS within 30 days of initial ED presentation. Most (five out of six) diagnosis of ACS in those who left at their own risk occurred within 7 days of the index presentation.

## 4 Discussion

We describe outcomes for a large cohort of ED chest pain presentations in WA using robust linked data. Overall, the age and sex ratio of our cohort are comparable to the WA population [16]. The proportion Indigenous is slightly higher than in the WA population (3.9% vs. 3.3%) [17]. The proportion of out‐of‐hours presentations is consistent with previously published values [1]. Most patients appropriately received a triage score of 2.

ED performance appeared efficient, with average time from presentation to first troponin of 49.7 min. An average time between first and second troponin of 2.5 h is consistent with use of modern hsTnI. Length of stay was in line with ACEM's Hospital Access Targets (Australia) for both admitted and discharge patients when considering those with a length of stay of less than 4 h and less than 8 h, but not for those with a length of stay less than 12 h [18].

We found 30‐day composite outcome of ACS/death for those discharged home from the ED was 0.4%, with a 30‐day death rate of 0.1%. Even discounting those whose discharge may have been in accordance with goals of care or deaths due to unrelated causes, this is likely an acceptable ‘miss‐rate’ for many emergency clinicians [19]. It is important to note that further attempts to reduce miss‐rate in low‐risk populations may cause increased harm through over investigation [19]. Outcomes for those discharged home in our cohort are also similar to those previously reported [20, 21]. In a systematic review and meta‐analysis of the HEART score, Van Den Berg and Body reported a pooled incidence of major adverse cardiac events (MACE) of 1.6% for low‐risk patients that were suitable for early discharge (with a mean of 6 weeks' follow‐up) [16]. However, Meek et al. report a lower 30‐day MACE of 0.09% in an Australian cohort of 1143 low‐risk chest pain patients who were discharged home from ED [21].

Patients who leave EDs against medical advice may be at increased risk of death and have higher readmission rates compared to those who are discharged home from ED [22, 23]. Those who ‘left at own risk’ made up a small proportion (0.5%) of the total cohort. All recorded events within the 30‐day follow up period were NSTEMIs with no deaths. Most events occurred within the first 7 days following discharge. Though absolute number of outcomes were small, 30‐day outcomes for those who ‘left at own risk’ were less than the overall cohort but higher than those discharged from ED. Out‐of‐hours presentations had a higher rate of 30‐day outcomes than in‐hours (7.1% vs. 6.6%, *p*‐value 0.01). This contrasts with Rolls et al. who found after‐hours presentation associated with lower 30‐day mortality in a large database study of ED presentations in Queensland, Australia [7].

### 4.1 Sex‐Based Differences

There was a statistically significant relationship between sex and triage scores, though a similar proportion of male and female patients received a triage score of 2, which is the recommended triage score from ACEM for ‘cardiac‐sounding’ chest pain. This relationship remained following propensity matching. Males with a triage score of 1, 2 or 3 were more likely to have a 30‐day outcome than females with the same triage score. This likely reflects the fact that there were more outcomes in males than females. Sex‐specific differences in outcomes for patients with ACS have been previously described [24, 25, 26, 27]. Kaul et al. report that women with ACS or chest pain were less likely than men to be admitted to or receive coronary revascularisation procedures, however there were no sex‐specific differences in 1‐year mortality [25]. Bugiardini et al. and found that women with STEMI have a higher risk of 30‐day death, likely due to delays in hospital presentation [26]. Stehli et al. also found higher 30‐day death in women with STEMI compared to men [27]. However, outcomes for undifferentiated chest pain presentations stratified by initial ED triage score have not previously been reported for Australian cohorts.

### 4.2 Representation to ED With Chest Pain

ED re‐presentations may reflect disease progression and appropriate safety netting (clear discharge instructions) or physician request. However, it is likely that many re‐presentations are potentially preventable [28]. We found 6.4% of patients had re‐presented to ED within 1 week of their initial presentation, and 14.8% within 30 days of their initial presentation. This is slightly higher than previous findings from Robinson et al., who report a re‐presentation of 4.9% within 72 h of ED discharge (single centre), and Dinh et al. who found 4.9% had re‐presented within 3 days (retrospective analysis of linked population registry) [28, 29].

## 5 Strengths and Limitations

A strength of this study is that we considered undifferentiated chest pain presentations at the point of triage. The relative geographical isolation of Perth also may reduce loss to follow‐up. The use of statewide linked administrative health data meant that we were able to capture all admissions to public and private hospitals in the state. This is a retrospective cohort study and so is reliant on the quality of coded data. While death is fully captured from linked state death registrations, recording of comorbidities may be incomplete. The dataset includes only WA data, meaning that if a patient presented and then left the state, any outcomes would not be captured, although this is likely to be low. The data used in this study is now more than 5 years old, and so may not be as generalisable in the context of decreased hospital access which has been experienced in the last 5 years.

Patients also may have presented with a symptom other than chest pain and have been subsequently investigated and diagnosed as MI. Alternatively, patients may have been included in our cohort but never investigated for a cardiac cause of chest pain. Including ‘Shortness of Breath’ as a presenting symptom is likely to capture many presentations that were not cardiac (which may explain why over half of presentations did not have troponin testing). However, myocardial ischaemia may present more commonly as breathlessness in women and the elderly [30, 31]. Time from symptom onset to first troponin is not available in this data. This may explain why a second troponin test was rarely positive if the first troponin test result was negative. Chest pain pathways may also have differed between hospitals, or changed over the study period.

To reduce bias and ensure independence of observations, only the index presentation was considered for each patient. Patients who present frequently to EDs may have a different risk profile. Finally, the complete medical notes for each individual presentation were not reviewed. ACS or death within 30 days may be unrelated to initial ED presentation. Death within 30 days may be expected and reflect appropriate management in some circumstances (e.g., palliative care). Our discharge home cohort is also not explicitly ‘low risk’, which limits comparisons to existing literature.

## 6 Conclusion

It was rare for patients who were discharged home from ED to subsequently be diagnosed with ACS within 30 days of ED discharge (0.4%). Additionally, 30‐day mortality for those discharged home from ED was low (0.1%). We found males were significantly more likely to receive a triage score of 1 than females. Males were also significantly more likely than females to have a 30‐day outcome. These differences in triage score allocation and outcomes were unchanged following propensity matching. However, there were no differences in triage score between males and females when considering a propensity matched cohort of only those who experienced 30‐day outcome. No patients ‘who did not wait’ or ‘left at their own risk’ died within 30 days. Discharge within 4 h was in line with ACEM guidance for both admitted and discharged patients. Overall, outcomes for chest pain presentations to EDs in WA are in line with international literature.

## Funding

This project was supported by the Western Australian Health Translation Network and the Australian Government's Medical Research Future Fund (MRFF) as part of the Rapid Applied Research Translation program. Authors who received grant: G.D. Funder Website: https://wahtn.org/ The funders had no role in study design, data collection and analysis, decision to publish, or preparation of the manuscript.

## Ethics Statement

This project received ethics and governance approval from the WA Department of Health Human Research Ethics Committee and Research Governance Office (RGS0000002876) and the Human Research Ethics Committee of The University of Western Australia (2021/ET000538).

## Conflicts of Interest

The authors declare no conflicts of interest.

## Supporting information


**Table S1:** Study cohort outcomes (within 7 days of index visit discharge).
**Appendix S1:** Comorbidities (ICD‐10‐AM Codes).
**Appendix S2:** Chest pain presenting symptom (from ED Dataset).
**Appendix S3:** Outcome definitions (ICD‐10‐AM code).
**Appendix S4:** Details of propensity matching.

## Data Availability

The authors have nothing to report.
