# Supplementary material for: Short‐Term Outcomes and Sex‐Based Analysis Following Chest Pain Presentations to Emergency Departments in Western Australia—An AUS‐MOCHA Substudy
Source: Emerg Med Australas. 2026 Feb 19;38(1):e70235. doi: 10.1111/1742-6723.70235 (PMC12921382; doi:10.1111/1742-6723.70235)
Supplement: Supplementary file 1 — Table S1: Study cohort outcomes (within 7 days of index visit discharge). Appendix S1: Comorbidities (ICD‐10‐AM Codes). Appendix S2: Chest pain presenting symptom (from ED Dataset). Appendix S3: Outcome definitions (ICD‐10‐AM code). Appendix S4: Details of propensity matching. [file EMM-38-0-s001.docx]

**Supplementary Table 1 – Study cohort Outcomes (Within 7 days of index visit discharge)**

Count (percent of sub cohort)

| Outcome | Total cohort (n=64404) | Admitted  (n=33572) | Discharged home (n=28087) | Did not wait/Left at own risk (n=345) |
| --- | --- | --- | --- | --- |
| ACS or Death | 4046 (6.3) | 3690 (11.0) | 57 (0.20) | 5 (1.45) |
| Death | 218 (0.3) | 172 (0.51) | 9 (0.03) | 0 (0) |
| ACS | 3959 (6.2) | 3608 (10.8) | 53 (0.2) | 5 (1.5) |
| STEMI | 852 (1.3) | 680 (2.0) | 4 (0.0) | 0 (0) |
| NSTEMI | 2241 (3.5) | 2117 (6.3) | 26 (0.1) | 5 (1.5) |
| Unstable Angina | 888 (1.4) | 831 (2.5) | 23 (0.1) | 0 (0) |
| Represented to ED | 4091 (6.4) | 2094 (6.2) | 1594 (5.7) | 46 (13.2) |
| Represented to ED with chest pain | 843 (1.3) | 415 (1.2) | 362 (1.3) | 6 (1.7) |

Cohort outcomes within 7 days of discharge from index visit according to discharge location.

ACS, acute coronary syndrome; STEMI, ST-segment elevation myocardial infarction; NSTEMI, non-ST-segment elevation myocardial infarction.

ACS = diagnosis of STEMI, or NSTEMI, or ACS (see appendix 3)

Transfer to another ED is not counted as representation

Representation with chest pain is based on presenting symptom at subsequent ED presentation

Within x days of index visit means date of outcome ≤ 7 days from time of index visit ED discharge

Counts of admitted, discharged, and left at own risk do not add up to count of total cohort as there are also other discharge destinations not included in this table (Died = 18, Unknown/other = 2382).

Departure destination “Home” includes discharge back to nursing home/hostel

Departure destination “Admitted” includes admission to ward, mental health unit, ED observation ward, or transfer to other hospital.

Departure destinations for cohort “Did not wait” n = 57 and “Left at own risk” n = 288

**Supplementary Table 2 - Length of stay in Emergency Department**

|  | Total cohort  (n=64404) | Admitted  (n=33572) | Discharged home (n=28087) | Did not wait/Left at own risk (n=345) |
| --- | --- | --- | --- | --- |
| Mean length of stay (hours) | 3.7 | 4.1 | 3.2 | 2.7 |
| **Length of stay (count (percent))** |  |  |  |  |
| <4 hours | 45481 (70.6) | 21124 (62.9) y | 22224 (79.1) n | 269 (78.0) |
| <6 hours | 57468 (89.2) | 28596 (85.2) y | 26400 (94.0) n/a | 321 (93.0) |
| <8 hours | 61532 (95.5) | 31365 (93.4) y | 27558 (98.1) y | 335 (97.1) |
| <12 hours | 63535 (98.7) | 32863 (97.9) n | 27968 (99.6) n | 345 (100) |

Length of stay in emergency department, stratified by discharge location

y = in line with the Australasian College for Emergency Medicine’s (ACEM) Hospital Access Targets (Australia)

n = not in line with ACEM’s Hospital Access Targets (Australia)

Note: ACEM does not provide an Australian Hospital Access Target for length of stay <6 hours for those discharged home.

Departure destination “Home” includes discharge back to nursing home/hostel

Departure destination “Admitted” includes admission to ward, mental health unit, ED observation ward, or transfer to other hospital.

Counts of admitted, discharged, and left at own risk do not add up to count of total cohort as there are also other discharge destinations not included in this table (Died = 18, Unknown/other = 2382).

Departure destinations for cohort “Did not wait” n = 57 and “Left at own risk” n = 288

**Supplementary Appendix 1 –** **Comorbidities (ICD-10-AM Codes)**

| **Condition** | **ICD-10-AM Code** |
| --- | --- |
| Acute Coronary Syndrome (ACS) | I21, I20.0 |
| Chest pain | R07.0–R07.4 |
| Hypertension | I10-I15 |
| Atrial fibrillation (AF) | I48.0, I48.1, I48.2 |
| Heart failure | I50 |
| Cerebrovascular disease | I60-I69, G45-G46 |
| Chronic Obstructive Pulmonary disease (COPD) | J40-J47 |
| Chronic kidney failure | N18 |
| Diabetes mellitus | E10-E14 |

**Supplementary Appendix 2 –** **Chest pain presenting symptom (from ED Dataset)**

PAIN -> CHEST -> CENTRAL

PAIN -> CHEST -> LEFT SIDED

PAIN -> CHEST

RESPIRATORY -> SHORT OF BREATH

Chest Pain

CARDIAC / VASCULAR -> PALPITATIONS/ ?ARRHYTHMIA

PAIN -> CHEST -> RIGHT SIDED

Pain Chest

PAIN -> ABDOMINAL -> EPIGASTRIC

CARDIAC / VASCULAR -> PALPITATIONS/ ?ARRHYTHMIA -> WITH CHEST PAIN

Short of Breath

CARDIAC / VASCULAR -> PALPITATIONS/ ?ARRHYTHMIA -> WITH CHEST PAIN AND SOB

PROVISIONAL DIAGNOSIS -> CARDIAC / VASCULAR -> ? UNSTABLE ANGINA

PROVISIONAL DIAGNOSIS -> CARDIAC / VASCULAR -> ? ACUTE MYOCARDIAL INFARCTION

**Supplementary Appendix 3 -** **Outcome Definitions (ICD-10-AM code)**

| Outcome | ICD-10-AM Code(s) |
| --- | --- |
| ST-segment elevation myocardial infarction (STEMI) | I21.0-I21.3 |
| Non-ST-segment elevation myocardial infarction (NSTEMI | I21.4 |
| Unstable Angina | I20.0 |
| Acute coronary syndrome (ACS) | 121.0-121.9, I20.0 |
| Chest pain | R07.0-R07.4 |

**Supplementary Appendix 4 –** **Details of propensity matching.**

To reduce confounding by baseline covariates, we performed 1:1 propensity score matching between female and male patients. Propensity scores were estimated using a logistic regression model in which sex (female vs. male) was regressed on a set of pre-specified covariates: age, Indigenous identity, past history of diabetes, hypertension, chronic obstructive pulmonary disease (COPD), cerebrovascular disease, acute coronary syndrome (ACS), atrial fibrillation (AF), chest pain, heart failure, and chronic kidney disease (CKD). The resulting propensity score reflected each patient’s probability of being female given their baseline characteristics.

Matching was conducted using nearest neighbour matching without replacement, within a calliper of 0.02 on the propensity score scale. For each female patient (treated group), one male patient (control group) was selected whose propensity score fell within ±0.02 and was closest in absolute difference. Matched pairs were discarded if no suitable control was found within the calliper.

Covariate balance before and after matching was assessed using standardized mean differences (SMDs). An SMD less than 0.1 was considered indicative of acceptable balance. After matching, the matched sample was used for subsequent analyses of 30-day mortality outcomes, including unadjusted comparisons and adjusted logistic regression models.
